# Microstructural Analysis of the Human Scapula: Mandibular Bone Tissue Engineering Perspectives

**DOI:** 10.3390/jfb15120386

**Published:** 2024-12-20

**Authors:** Ilya L. Tsiklin, Denis S. Bezdenezhnych, Aleksei S. Mantsagov, Alexandr V. Kolsanov, Larisa T. Volova

**Affiliations:** 1 Biotechnology Research Institute, Samara State Medical University, 443079 Samara, Russia; 2 City Clinical Hospital Botkin, Moscow Healthcare Department, 125284 Moscow, Russia; 3 Samara Regional Bureau of Forensic Examination, 443079 Samara, Russia

**Keywords:** bone tissue engineering, biodegradable biopolymers, human scapula, mandible, allogenic bone scaffold, microstructural analysis, trabecular bone, cortical bone, micro-computed tomography (micro-CT), regenerative medicine

## Abstract

Mandibular bone defect reconstruction remains a significant challenge for surgeons worldwide. Among multiple biodegradable biopolymers, allogeneic bone scaffolds derived from human sources have been used as an alternative to autologous bone grafts, providing optimal conditions for cell recruitment, adhesion, and proliferation and demonstrating significant osteogenic properties. This study aims to investigate the bone microstructure of the human scapula as a source for allogeneic bone scaffold fabrication for mandibular tissue engineering purposes. We created color-coded anatomical maps of the scapula and the mandible, reflecting the best anatomical and geometrical match. In this pilot study, we hypothesized a microstructural similarity of these bone structures and evaluated the human scapula’s bone tissue engineering potential for mandibular bone tissue engineering by focusing on the microstructural characteristics. Lyophilized human scapular and mandibular bioimplants were manufactured and sterilized. Experimental bone samples from the scapula’s acromion, coracoid, and lateral border from the mandibular condyle, mandibular angle, and mental protuberance were harvested and analyzed using micro-CT and quantitative morphometric analysis. This pilot study demonstrates significant microstructural qualitative and quantitative intra-group differences in the scapular and mandibular experimental bone samples harvested from the various anatomical regions. The revealed microstructural similarity of the human scapular and mandibular bone samples, to a certain extent, supports the stated hypothesis and, thus, allows us to suggest the human scapula as an alternative off-the-shelf allogeneic scaffold for mandibular reconstruction and bone tissue engineering applications.

## 1. Introduction

Extensive mandibular bone defect reconstruction remains a significant challenge in oral and maxillofacial surgery [1,2,3].

Bone tissue engineering suggests a promising approach to efficient bone defect repair [4,5,6,7,8]. Allogeneic bone scaffolds derived from human sources demonstrate appropriate biomimetic, osteogenic, and mechanical properties, a high biodegradability rate, off-the-shelf availability, and a long shelf life [9,10,11,12,13,14,15]. Ethical issues of using the human mandible as an orthotopic allogenic bioimplant, challenging harvesting techniques and aesthetic concerns inspire the search for heterotopic human bone bioimplants for regeneration medicine purposes. Additive manufacturing techniques, such as individual 3D-printed cutting guides, plates, and bioimplants, enabled the designing of personalized patient-specific bone scaffolds. Despite recent significant advances in using natural and synthetic polymers and hydrogels for scaffold fabrication, the potential benefit from allogeneic human bone scaffolds demonstrating native bone properties could be particularly valuable [16,17,18,19,20].

The human scapula, as a unique part of the appendicular skeleton presented with a complex anatomical shape and contributes to various mesoderm, ectoderm, and neural crest derivatives during its embryonic development, has been widely used as a free vascularized flap for mandible defect reconstruction. The scapular flap is particularly beneficial in midface and mandible body reconstruction due to its anatomical shape. Furthermore, donor-site morbidity and postoperative concerns of the scapular flap are generally less significant comparing fibular or iliac crest bone flaps. Typically, the bone flap volume that can be safely harvested from the scapula in a living person is limited to the lateral scapular border and, in some cases, to the scapular angle [21,22,23,24,25]. The potential of the human scapula as a heterotopic allogenic bone scaffold for mandible reconstruction has not been previously explored. The anatomical shape similarity of the scapular coracoid process and the mandibular condyle inspired us to investigate their trabecular and cortical bone microstructure and to hypothesize their microstructural similarity. Other scapular bone structures, such as the lateral border and acromion, could also be considered potential allogeneic bone bioimplants and scaffolds for mandibular bone tissue engineering. Several studies on the scapula and the mandible morphology and microstructural analysis reported high-resolution micro-computed tomography (micro-CT) as an effective, non-destructive method to assess scapular trabecular and cortical bone morphology and vascularization [26,27,28,29,30,31,32,33,34,35,36,37,38]. This perspective underscores the need for further research and development in mandible reconstruction and bone tissue engineering, a field with significant potential for improving patient outcomes.

This pilot study evaluates the human scapula’s bone tissue engineering potential for mandible defect repair by focusing on the microstructural characteristics.

## 2. Materials and Methods

### 2.1. Manufacture of the Scapular and Mandibular Bioimplants Lyoplast^®^

**Figure 1:**
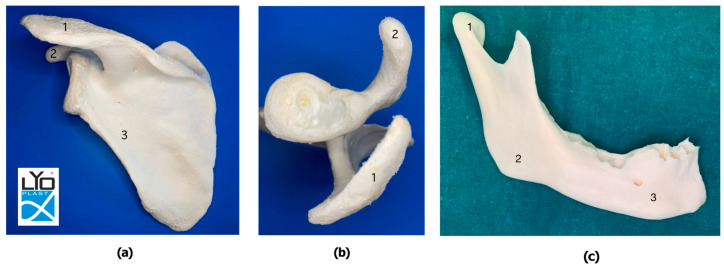
Lyophilized left scapula (**a**,**b**) bioimplant Lyoplast^®^ ((**a**)—posterior view, (**b**)—lateral view: 1—scapular acromion process (SAP); 2—scapular coracoid process (SCP); 3—scapular lateral border (SLB)) and right hemimandible (**c**) bioimplant Lyoplast^®^: 1—mandibular condyle (MC); 2—mandibular angle (MA); 3—mental protuberance (MP).

Scapular and mandibular bone bioimplants Lyoplast^®^ were manufactured at the Samara tissue bank at the “BioTech” Biotechnology Center, Samara State Medical University (RF patent No. 2366173 of 15 May 2008; reg. No. RU CMS-RU.PT02.00115). The study was conducted according to the guidelines of the Declaration of Helsinki. Ethical approval by the Committee on Bioethics of Samara State Medical University (protocol extract No.215; 20.01.2021). Six scapulas (three right and three left) and three mandibles were harvested from six middle-aged male cadavers without a history of osteoporosis, scapula or mandible trauma, and previous relevant surgical procedures (Regional Samara Bureau of Forensic Examination, Samara, Russia; Patent RF № 2366173 of 15 May 2008. “Method of manufacturing large-block lyophilized bone implants” Volova L.T.). After compulsory mechanical and low-frequency ultrasonic treatment with an ultrasonic bath with a frequency of 24–40 kHz (“Sapphire” TTC (RMD) LTD, Moscow, Russia), lyophilization (vacuum drying by sublimation) of the bone samples was performed (sublimation unit ALPHA2-4LSC; Martin Christ Gefriertrocknungsanlagen GmbH, Osterode am Harz, Germany). Scapula samples were sterilized by gamma rays (GU-200 M; NIIP Joint-Stock Company, Moscow, Russia). A spectrophotometer was used for the estimation of the residual content of lipids in the bone samples (SF-56 “Lomo-Spektr”, St. Petersburg, Russia). A thermogravimetric infrared moisture meter was used to determine the humidity of the final bone samples (Sartorius-MA-150, Malente, Germany), as shown in Figure 1.

### 2.2. Anatomical Mapping of the Human Scapula

Developing the “anatomical map” of the human scapula as the potential source of an allogeneic bone scaffold and bone bioimplant involves identifying the main anatomical regions with the best anatomical and geometrical match to the mandible and presumably different morpho-structural properties, followed by microstructural analysis of experimental bone samples harvested from these regions.

**Figure 2:**
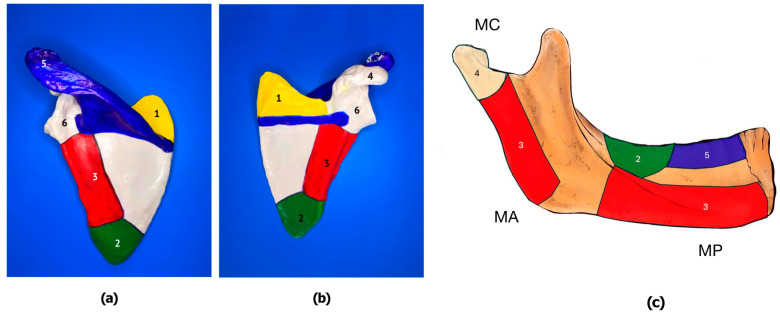
Anatomical mapping of the human scapula ((**a**)—posterior view; (**b**)—anterior view; 1—superior angle, 2—inferior angle, 3—lateral border, 4—coracoid process, 5—acromion, 6—glenoid) and the human mandible (**c**) with respective color-coded recipient zones: mandibular condyle (MC); mandibular angle (MA); mental protuberance (MP).

Developing the “anatomical map” of the human mandible is based on identifying potential donor sites for mandible reconstruction (due to the bone atrophy, resection, or trauma) with presumably different morpho-structural properties that potentially match particular scapular areas as the sources of the donor bone tissue, as shown in Figure 2.

### 2.3. Preparation of the Experimental Scapular Bone Samples for mCT Scanning

**Figure 3:**
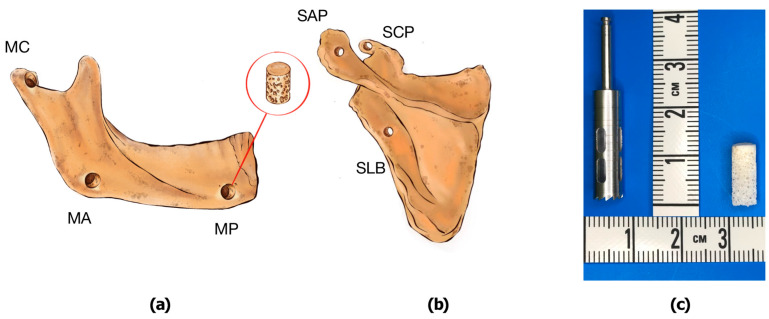
Preparation of the experimental mandibular and scapular bone samples ((**a**)—mandibular condyle (MC), mandibular angle (MA), mental protuberance (MP); (**b**)—scapular coracoid process (SCP), scapular acromion process (SAP), scapular lateral border (SLB); (**c**)—trepan bur and harvested experimental bone sample)).

Eighteen scapular cylinder-shaped experimental bone samples (six from the lateral scapular border, six from the scapular coracoid process, and six from the scapular acromion process) and eighteen mandibular cylinder-shaped experimental bone samples (six from the mandibular condyle, six from the mandibular angle, and six from the mandibular mental protuberance) with a diameter of 5 mm were obtained using trepan bur with an inner diameter of 5 mm. The height of the bone samples varied from 7.5 to 11.5 mm with a mean of 10.3 ± 0.49 mm. Each sample included at least one cortical plate and trabecular bone fragment, as shown in Figure 3.

### 2.4. Micro-Computed Tomography of the Experimental Bone Samples

**Figure 4:**
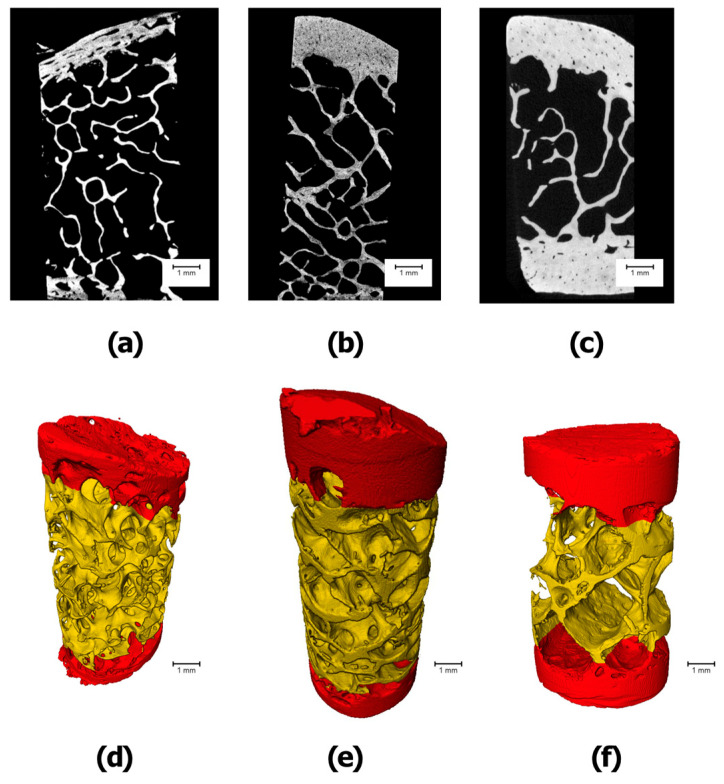
Scapular bone samples’ image acquisition, volume rendering, and segmentation ((**a**,**d**)—SCP; (**b**,**e**)—SAP; (**c**,**f**)—SLB)).

**Figure 5:**
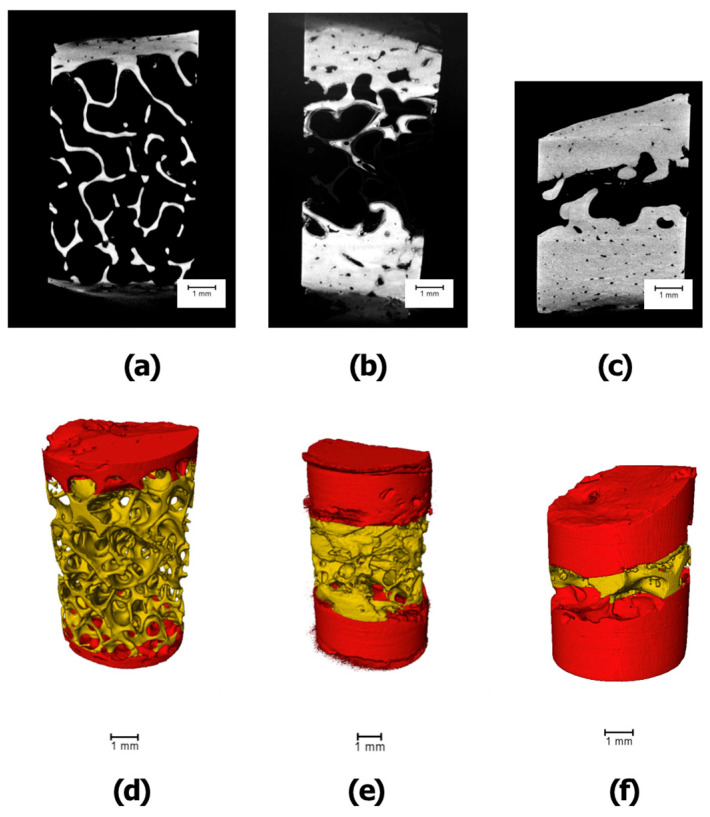
Mandibular bone samples’ image acquisition, volume rendering, and segmentation ((**a**,**d**)—SCP; (**b**,**e**)—SAP; (**c**,**f**)—SLB)).

Micro-CT scanning of the lyophilized scapular and mandibular bone samples was performed in the Laboratory of Microanalysis in Skolkovo Technopark (Moscow, Russia). The high-resolution 3D X-ray microscope VersaXRM-500 (Xradia, Inc., Pleasanton, CA, USA) with voltage range 30–160 kV, maximum power 10 W, 360° rotation, and maximum spatial resolution of <0.7–1 µm (True Spatial Resolution™) was used. In the first stage, the scanning of the sample was performed using a resolution of 8.6 µm/pixel at a voltage of 80 kV with a set of 1081 projections and 0.5 s exposition time. The obtained data were reconstructed with the Filtered Back Projection method and a segmentation of the cortical and trabecular bone was performed, as shown in Figure 4 and Figure 5.

### 2.5. Morphometric Analysis of the Bone Samples

Visual inspection of the 3D-rendered and segmented experimental scapular and mandibular bone samples was followed by their analysis, including evaluation of the trabecular and cortical bone volumetric parameters and trabecular bone connectivity. The bone morphometric analysis was performed using AnylizeDirect software (AnylizeDirect 12.0, Inc., Stilwell, KS, USA) and PerGeos software (version 2022.1, Thermo Fisher Scientific Inc., Waltham, MA, USA).

Bone morphometric parameters analyzed in this study are summarized in Table 1.

### 2.6. Statistical Analysis

Statistical analysis was performed using the RStudio software package (Version 4.3.1; RStudio, Boston, MA, USA). Descriptive statistics included calculation of the mean and standard deviation. The Shapiro–Wilk test was used to check for the distribution normality of the data. Differences in parameters between the bone samples were tested using the Levene test, two-sample *t*-test, one-way ANOVA, and Kruskal–Wallis tests with multiple comparisons (Tukey’s test) at the level of significance of α = 0.05.

## 3. Results

### 3.1. Results of the Morphometric Microstructural Analysis of the Scapular Bone Samples

**Figure 6:**
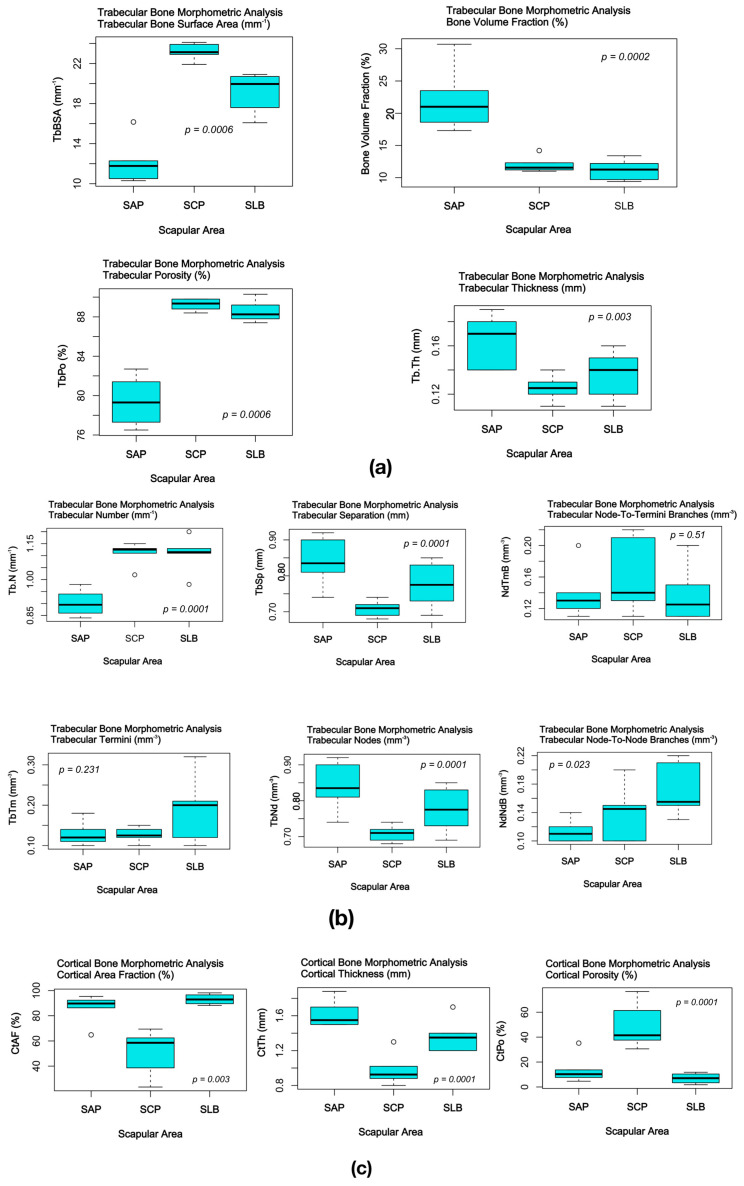
Comparative analysis of the trabecular and cortical bone morphometric parameters of the experimental scapular bone samples: (**a**) trabecular bone volumetric parameters; (**b**) trabecular bone connectivity parameters; (**c**) cortical bone parameters.

Morphometric parameters of the experimental scapular bone samples are summarized in Table 2. Graphical interpretation of the scapular bone samples comparative microstructural analysis (one-way Analysis of Variance (ANOVA) with Tukey multiple comparison test) is presented in Figure 6.

**Figure 7:**
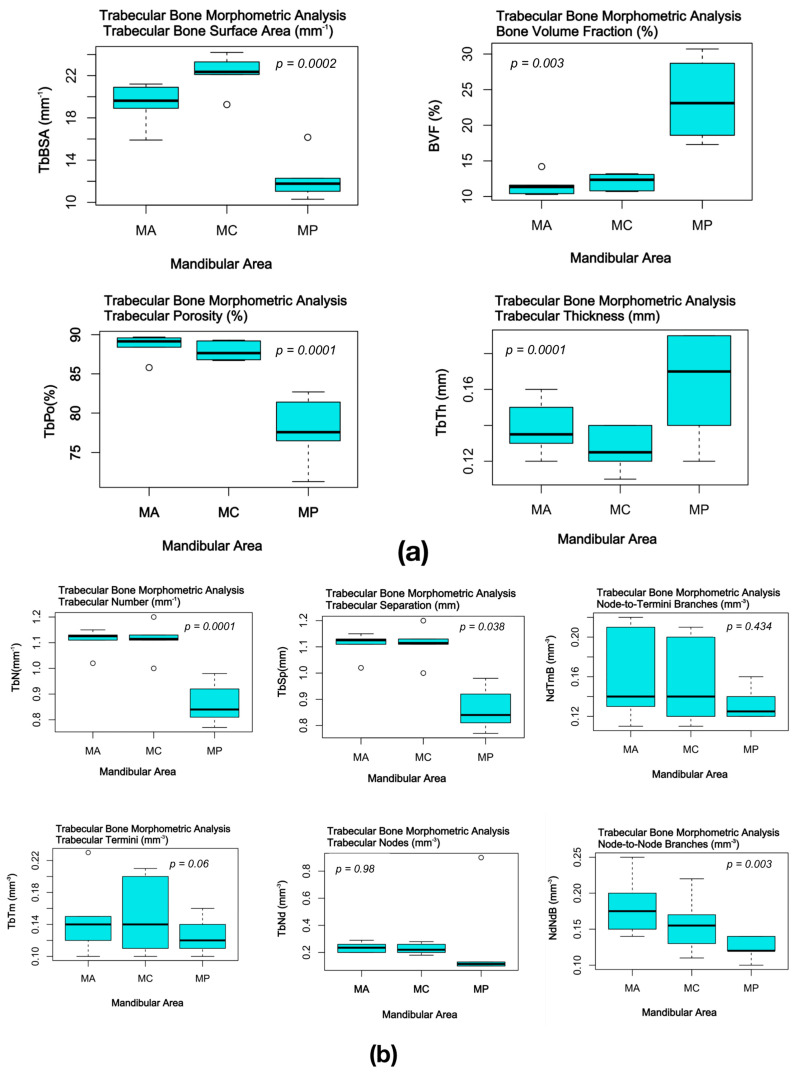

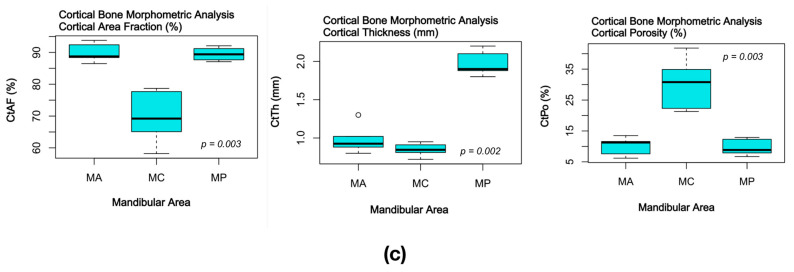
Comparative analysis of the trabecular and cortical bone morphometric parameters of the experimental mandibular bone samples: (**a**) trabecular bone volumetric parameters; (**b**) trabecular bone connectivity parameters; (**c**) cortical bone parameters.

Morphometric parameters of the experimental mandibular bone samples are summarized in Table 3. Graphical interpretation of the scapular bone samples comparative microstructural analysis is presented in Figure 7.

### 3.2. Results of the Comparative Microstructural Analysis of the Scapular and Mandibular Experimental Bone Samples

#### 3.2.1. Comparative Analysis of the Scapular Coracoid Process and Mandibular Condyle Bone Samples

There was no statistically significant difference in trabecular and cortical bone microstructure and trabecular connectivity parameters of the scapular coracoid process and mandibular condyle experimental bone samples except the Tb.Po parameter demonstrating a marginal significance (Table 4). Graphical interpretation of the comparative analysis is presented in the Supplementary Materials (Figure S1).

#### 3.2.2. Comparative Analysis of the Scapular Lateral Border and Mandibular Angle Bone Samples

There was no statistically significant difference in trabecular bone microstructure and trabecular connectivity parameters of the scapular lateral border and mandibular angle experimental bone samples. Cortical thickness was significantly higher in the scapular lateral border bone samples, while cortical porosity was higher in the mandibular angle bone samples (Table 5). Graphical interpretation of the comparative analysis is presented in the Supplementary Materials (Figure S2).

#### 3.2.3. Comparative Analysis of the Scapular Lateral Border and Mandibular Mental Protuberance Bone Samples

Most of the trabecular bone parameters of the scapular lateral border and the mandibular mental protuberance demonstrated statistically significant differences. Trabecular thickness was similar for both bone samples, and some of the trabecular connectivity parameters (Tb.Sp, Tb.Tm, NdTmB) had no statistically significant difference. Cortical thickness was significantly higher in the mental protuberance bone samples, while cortical porosity was similar for both bone samples (Table 6). Graphical interpretation of the comparative analysis is presented in the Supplementary Materials (Figure S3).

#### 3.2.4. Comparative Analysis of the Scapular Acromion and Mandibular Angle Bone Samples

The volumetric trabecular bone parameters (Tb.BSA, BVF, Tb.Po, Tb.Th) of the scapular acromion process and the mandibular angle demonstrated statistically significant differences. Some trabecular connectivity parameters (Tb.N, Tb.Nd, NdNdB) also showed statistically significant differences. Cortical thickness was significantly higher in the scapular acromion bone samples, while cortical porosity was similar for both bone samples (Table 7). Graphical interpretation of the comparative analysis is presented in Supplementary Materials (Figure S4).

#### 3.2.5. Comparative Analysis of the Scapular Acromion and Mental Protuberance Bone Samples

There was no statistically significant difference in trabecular bone microstructure and trabecular connectivity parameters of the scapular acromion process and mandibular mental protuberance experimental bone samples. Cortical thickness was significantly higher in the mandibular mental protuberance bone samples, while there was no statistically significant difference in cortical porosity of the bone samples (Table 8). Graphical interpretation of the comparative analysis is presented in Supplementary Materials (Figure S5).

## 4. Discussion

Our preliminary two-dimensional evaluation of the bone samples using mCT, followed by 3D rendering and segmentation, enabled us to visualize distinct qualitative intra-group microstructural differences in the trabecular and cortical bone architecture of the scapular and mandibular bone samples. This process instills confidence in the reliability of our finding. Among the scapular bone samples, we observed visually higher trabecular and cortical thickness in the scapular acromion and scapular lateral border samples compared to the coracoid process. Additionally, the trabecular structure of the coracoid process appeared to exhibit higher trabecular connectivity. The mandibular mental protuberance showed significantly thicker trabecular and cortical bone than the mandibular condyle and mandibular angle bone samples.

The bone morphometric comparative analysis, which employed a one-way ANOVA with multiple comparisons, further supported and instilled confidence in the reliability of our findings. This statistical method revealed significant quantitative intra-group differences in the majority of the trabecular and cortical microstructural parameters of the scapular and mandibular bone samples. Interestingly, despite the similar function of the scapular acromion and coracoid processes and similar embryonic developmental characteristics, we did not find microstructural similarity of their trabecular and cortical bone architecture apart from some of the trabecular connectivity parameters that demonstrated no significant difference. Moreover, considering different sources of embryonic development, we did not reveal any significant microstructural difference between the coracoid process and the scapular lateral border.

The results of the presented above inter-group microstructural comparisons strongly supported the hypothesis on the potential microstructural similarity in the following three pairs of the scapular and the mandibular experimental bone samples:

- SCP-MC (presented with the high similarity of the trabecular and cortical bone microarchitecture parameters);
- SLB-MA (presented with the high similarity of the trabecular bone microarchitecture parameters; however, the cortical bone microstructure showed a significant difference);
- SAP-MP (presented with the high similarity of the trabecular and cortical bone microarchitecture parameters)

The following two pairs of the scapular and the mandibular experimental bone samples did not show any evidence of microstructural similarity:

- SLB-MP (most trabecular and cortical bone microarchitecture parameters were significantly different);
- SAP-MA (most trabecular and cortical bone microarchitecture parameters were significantly different).

Incorporation of the bone microarchitecture parameters into the virtual surgical planning workflow alongside the anatomical maps of the mandible and the scapula can become a simple and comprehensive tool, improving the virtual planning of the patient-specific bone scaffold depending on particular clinical needs. Apart from the optimal geometrical match of the bone scaffold, the bone microarchitectural similarity of the donor and recipient sites may potentially improve the quality and predictability of the bone reconstruction procedure.

## 5. Conclusions

This pilot study demonstrates significant microstructural qualitative and quantitative intra-group differences in the scapular and mandibular experimental bone samples harvested from the various anatomical regions.

By creating anatomical maps, we emphasized the best correspondence of the various scapular and mandibular bone structures and hypothesized that their structural anatomical shape similarity might be reflected at the microstructural level. To a certain extent, the revealed microstructural similarity of the human scapular and mandibular bone samples supports this hypothesis. It allows us to suggest the human scapula as an alternative off-the-shelf allogeneic scaffold for mandibular reconstruction and bone tissue engineering applications.

Further analysis of the scapular and mandibular bone structure based on a larger sample size could be reasonable to support or reject the hypothesis of their microstructural similarity entirely.

Additive manufacturing of personalized allogenic heterotopic scapular bone scaffolds and bioimplants, considering the microstructural matching criteria between the mandibular bone defect and the bone scaffold, is a crucial next step in this research, underscoring the need for further investigation.

## 6. Patents

Patent RF № 2170016 from 17 February 1999 “Method of saturation of bone spongy tissue grafts with medication” Volova L.T., Kirilenko A.G., Uvarovsky B.B.
Patent RF № 2156139 from 15 March 1999 “Method of sterilization of lyophilized bone transplants” Volova L.T., Kirilenko A.G., Uvarovsky B.B.
Patent RF № 99108699 from 21 April 1999 “Method of bone marrow removal from cancellous bone grafts” Volova L.T., Kirilenko A.G.
Patent RF № 2366173 of 15 May 2008. “Method of manufacturing large-block lyophilized bone implants” Volova L.T.

## Figures and Tables

**Table 1:** Morphometric parameters of the trabecular and cortical bone.

**Trabecular Bone** **Morphometric Parameters**	**Abbreviation**	**Description**	**Unit**
Total Volume	TV	Total volume of the bone sample	mm^3^
Bone Volume	BV	Volume of bone per sample volume	mm^3^
Bone Volume Fraction	BVF	BVF = BV/TV × 100	%
Bone Surface	BS	Bone surface per sample	mm^2^
Trabecular Porosity	Tb.Po	Tb.Po = 1 − BV/TV	%
Bone Surface Density	BSD	BSD = BS/TV	mm^−1^
Trabecular Bone Surface Area	Tb.BSA	Tb.BSA = BS/BV	mm^−1^
Trabecular Number	Tb.N	The inverse of the mean distance between the midlines of the trabeculae Tb.N = BV:TV/Tb.Th	mm^−1^
Trabecular Thickness	Tb.Th	The mean trabecular bone thickness/Tb.Th = 2/BS:BV	mm
Trabecular Separation	Tb.Sp	The mean distance between the trabeculae/Tb.Sp = 1/Tb.N − Tb.Th	mm
Trabecular Nodes	Tb.Nd	The number of trabecular intersections per bone volume/Tb.Nd = Nd/BV	mm^−3^
Trabecular Termini	Tb.Tm	The number of trabecular terminal branches per bone volume/Tb.Tm = Tm/BV	mm^−3^
Node-to-Termini Ratio	NdTm R	NdTm R = Nd/Tm	n
Node-to-Node Branches	NdNdB	The number of branches between trabecular intersections per bone volume/NdNdB = NdNd/BV	mm^−3^
Node-to-Termini Branches	NdTmB	The number of branches between trabecular intersections and terminal branches per bone volume/NdTmB = NdTm/BV	mm^−3^
**Cortical Bone** **Morphometric Parameters**	**Abbreviation**	**Description**	**Unit**
Total Cross-sectional Area	Tt.Ar.	Total cross-sectional surface of the cortical bone	mm^2^
Cortical Bone Area	Ct.Ar.	Cortical bone surface	mm^2^
Cortical Area Fraction	Ct.A.F	Ct.A.F = Ct.Ar/Tt.Ar × 100	%
Average Cortical Thickness	Ct.Th	The mean of cortical bone thickness	mm
Cortical Porosity	Ct.Po	Ct.Po = 1 − Ct.Ar/Tt.Ar × 100	%

**Table 2:** Trabecular and cortical bone morphometric parameters of the experimental scapular bone samples.

**Trabecular Bone** **Morphometric Parameters**	**SCP (n = 6)** **Mean ± SD**	**SAP (n = 6)** **Mean ± SD**	**SLB (n = 6)** **Mean ± SD**	**One-Way ANOVA** ***p*-Value**
Tb.BSA (mm^−1^)	23.17 ± 1.78	12.13 ± 2.12	19.2 ± 1.94	0.0006 ***
BVF (%)	11.95 ± 0.18	22.01± 4.88	11.2 ± 1.53	0.0002 ***
Tb.Po (%)	89.25 ± 0.59	79.41 ± 2.51	88.53 ± 1.05	0.0006 ***
Tb.Th (mm)	0.12 ± 0.01	0.16 ± 0.02	0.14 ± 0.02	0.003 **
Tb.N (mm^−1^)	1.11 ± 0.04	0.9 ± 0.04	1.11 ± 0.07	0.0001 ***
Tb.Sp (mm)	0.70 ± 0.02	0.84 ± 0.06	0.77 ± 0.06	0.0001 ***
Tb.Nd (mm^−3^)	0.20 ± 0.04	0.12 ± 0.01	0.26 ± 0.05	0.0001 ***
Tb.Tm (mm^−3^)	0.12 ± 0.02	0.13 ± 0.03	0.19 ± 0.08	0.23
NdNdB (mm^−3^)	0.14 ± 0.04	0.11 ± 0.02	0.17 ± 0.04	0.023 *
NdTmB (mm^−3^)	0.16 ± 0.05	0.14 ± 0.03	0.13 ± 0.03	0.51
**Cortical Bone** **Morphometric Parameters**	**SCP (n = 6)** **Mean ± SD**	**SAP (n = 6)** **Mean ± SD**	**SLB (n = 6)** **Mean ± SD**	**One-Way ANOVA** ***p*-value**
Ct.A.F (%)	51.8 ± 1.73	86.4 ± 11.3	93.1 ± 3.9	0.003 **
Ct.Th (mm)	0.98 ± 0.18	1.61± 0.15	1.36 ± 0.18	0.0001 ***
Ct.Po (%)	48.2 ± 1.73	13.6 ± 11.1	6.9 ± 3.9	0.0001 ***

Level of significance α = 0.05 (* *p*-value < 0.05; ** *p*-value < 0.001; *** *p*-value < 0.0001). SD—Standard Deviation.

**Table 3:** Trabecular and cortical bone morphometric parameters of the experimental mandibular bone samples.

**Trabecular Bone** **Morphometric Parameters**	**MC (n = 6)** **Mean ± SD**	**MA (n = 6)** **Mean ± SD**	**MP (n = 6)** **Mean ± SD**	**One-Way ANOVA** ***p*-Value**
Tb.BSA (mm^−1^)	22.26 ± 1.66	19.35 ± 1.93	12.22 ± 2.04	0.0002 ***
BVF (%)	12.08 ± 1.09	11.53 ± 1.41	23.6 ± 5.32	0.003 ***
Tb.Po (%)	87.9 ± 1.13	88.63 ± 1.47	77.85 ± 4.02	0.0001 ***
Tb.Th (mm)	0.12 ± 0.01	0.14 ± 0.01	0.16 ± 0.02	0.0001 **
Tb.N (mm^−1^)	1.11 ± 0.06	1.1 ± 0.04	0.86 ± 0.06	0.0001 ***
Tb.Sp (mm)	0.72 ± 0.04	0.76 ± 0.07	0.81 ± 0.05	0.038 *
Tb.Nd (mm^−3^)	0.22 ± 0.04	0.24 ± 0.04	0.23 ± 0.03	0.98 *
Tb.Tm (mm^−3^)	0.15 ± 0.04	0.16 ± 0.04	0.12 ± 0.02	0.06
NdNdB (mm^−3^)	0.17 ± 0.03	0.18 ± 0.04	0.12 ± 0.02	0.003 **
NdTmB (mm^−3^)	0.15 ± 0.04	0.16 ± 0.04	0.13 ± 0.01	0.43
**Cortical Bone** **Morphometric Parameters**	**MC (n = 6)** **Mean ± SD**	**MA (n = 6)** **Mean ± SD**	**MP (n = 6)** **Mean ± SD**	**One-way ANOVA** ***p*-value**
Ct.A.F (%)	69.7 ± 7.73	89.7 ± 2.76	89.5 ± 2.05	0.003 **
Ct.Th (mm)	0.85 ± 0.18	0.98 ± 0.17	1.96 ± 0.15	0.002 **
Ct.Po (%)	30.3 ± 7.73	10.23 ± 2.75	9.6 ± 2.5	0.003 **

Level of significance α = 0.05 (* *p*-value < 0.05; ** *p*-value < 0.001; *** *p*-value < 0.0001). SD—Standard Deviation.

**Table 4:** Results of the comparative microstructural analysis of the trabecular and cortical bone morphometric parameters of the experimental scapular coracoid and mandibular condyle bone samples.

**Trabecular Bone** **Morphometric Parameters**	**SCP (n = 6)** **Mean ± SD**	**MC (n = 6)** **Mean ± SD**	**Two-Sample *t*-Test** ***p*-Value**
Tb.BSA (mm^−1^)	23.17 ± 1.78	22.26 ± 1.66	0.26
BVF (%)	11.95 ± 0.18	12.08 ± 1.09	0.86
Tb.Po (%)	89.25 ± 0.59	87.9 ± 1.13	0.032 *
Tb.Th (mm)	0.12 ± 0.01	0.12 ± 0.01	0.80
Tb.N (mm^−1^)	1.11 ± 0.04	1.11 ± 0.06	0.96
Tb.Sp (mm)	0.70 ± 0.02	0.72 ± 0.04	0.55
Tb.Nd (mm^−3^)	0.20 ± 0.04	0.22 ± 0.04	0.84
Tb.Tm (mm^−3^)	0.12 ± 0.02	0.15 ± 0.04	0.29
NdNdB (mm^−3^)	0.14 ± 0.04	0.17 ± 0.03	0.55
NdTmB (mm^−3^)	0.16 ± 0.05	0.15 ± 0.04	0.46
**Cortical Bone** **Morphometric Parameters**	**SCP (n = 6)** **Mean ± SD**	**MC (n = 6)** **Mean ± SD**	**Two-Sample *t*-test** ***p*-value**
Ct.A.F (%)	51.8 ± 1.73	69.7 ± 7.73	0.054
Ct.Th (mm)	0.98 ± 0.18	0.85 ± 0.18	0.14
Ct.Po (%)	48.2 ± 1.73	30.3 ± 7.73	0.054

Level of significance: α = 0.05 (* *p*-value < 0.05). SD—Standard Deviation.

**Table 5:** Results of the comparative microstructural analysis of the trabecular and cortical bone morphometric parameters of the experimental scapular lateral border and mandibular angle bone samples.

**Trabecular Bone** **Morphometric Parameters**	**SLB (n = 6)** **Mean ± SD**	**MA (n = 6)** **Mean ± SD**	**Two-Sample *t*-Test** ***p*-Value**
Tb.BSA (mm^−1^)	19.2 ± 1.94	19.35 ± 1.93	0.88
BVF (%)	11.2 ± 1.53	11.53 ± 1.41	0.70
Tb.Po (%)	88.53 ± 1.05	88.63 ± 1.47	0.89
Tb.Th (mm)	0.14 ± 0.02	0.14 ± 0.01	0.86
Tb.N (mm^−1^)	1.11 ± 0.07	1.1 ± 0.04	0.96
Tb.Sp (mm)	0.77 ± 0.06	0.76 ± 0.07	0.67
Tb.Nd (mm^−3^)	0.26 ± 0.05	0.24 ± 0.04	0.4
Tb.Tm (mm^−3^)	0.19 ± 0.08	0.16 ± 0.04	0.25
NdNdB (mm^−3^)	0.17 ± 0.04	0.18 ± 0.04	0.62
NdTmB (mm^−3^)	0.13 ± 0.03	0.16 ± 0.04	0.37
**Cortical Bone** **Morphometric Parameters**	**SLB (n = 6)** **Mean ± SD**	**MA (n = 6)** **Mean ± SD**	**Two-sample *t*-test** ***p*-value**
Ct.A.F (%)	93.1 ± 3.9	89.7 ± 2.76	0.0002 ***
Ct.Th (mm)	1.36 ± 0.18	0.98 ± 0.17	0.0004 ***
Ct.Po (%)	6.9 ± 3.9	10.23 ± 2.75	0.0002 ***

Level of significance: α = 0.05 (* *p*-value < 0.05; ** *p*-value < 0.001; *** *p*-value < 0.0001). SD—Standard Deviation.

**Table 6:** Results of the comparative microstructural analysis of the trabecular and cortical bone morphometric parameters of the experimental scapular lateral border and mandibular mental protuberance bone samples.

**Trabecular Bone** **Morphometric Parameters**	**SLB (n = 6)** **Mean ± SD**	**MP (n = 6)** **Mean ± SD**	**Two-Sample *t*-Test** ***p*-Value**
Tb.BSA (mm^−1^)	19.2 ± 1.94	12.22 ± 2.04	0.0002 ***
BVF (%)	11.2 ± 1.53	23.6 ± 5.32	0.0017 **
Tb.Po (%)	88.53 ± 1.05	77.85 ± 4.02	0.001 **
Tb.Th (mm)	0.14 ± 0.02	0.16 ± 0.02	0.09
Tb.N (mm^−1^)	1.11 ± 0.07	0.86 ± 0.06	0.0001 ***
Tb.Sp (mm)	0.77 ± 0.06	0.81 ± 0.05	0.24
Tb.Nd (mm^−3^)	0.26 ± 0.05	0.23 ± 0.03	0.0007 ***
Tb.Tm (mm^−3^)	0.19 ± 0.08	0.12 ± 0.02	0.09
NdNdB (mm^−3^)	0.17 ± 0.04	0.12 ± 0.02	0.024 *
NdTmB (mm^−3^)	0.13 ± 0.03	0.13 ± 0.01	0.75
**Cortical Bone** **Morphometric Parameters**	**SLB (n = 6)** **Mean ± SD**	**MA (n = 6)** **Mean ± SD**	**Two-sample *t*-test** ***p*-value**
Ct.A.F (%)	93.1 ± 3.9	89.5 ± 2.05	0.08
Ct.Th (mm)	1.36 ± 0.18	1.96 ± 0.15	0.0001 ***
Ct.Po (%)	6.9 ± 3.9	9.6 ± 2.5	0.08

Level of significance: α = 0.05 (* *p*-value < 0.05; ** *p*-value < 0.001; *** *p*-value < 0.0001). SD—Standard Deviation.

**Table 7:** Results of the comparative microstructural analysis of the trabecular and cortical bone morphometric parameters of the experimental scapular acromion and mandibular angle bone samples.

**Trabecular Bone** **Morphometric Parameters**	**SAP (n = 6)** **Mean ± SD**	**MA (n = 6)** **Mean ± SD**	**Two-Sample *t*-Test** ***p*-Value**
Tb.BSA (mm^−1^)	12.13 ± 2.12	19.35 ± 1.93	0.0001 ***
BVF (%)	22.01± 4.88	11.53 ± 1.41	0.002 **
Tb.Po (%)	79.41 ± 2.51	88.63 ± 1.47	0.0001 ***
Tb.Th (mm)	0.16 ± 0.02	0.14 ± 0.01	0.034 *
Tb.N (mm^−1^)	0.9 ± 0.04	1.1 ± 0.04	0.0001 ***
Tb.Sp (mm)	0.84 ± 0.06	0.76 ± 0.07	0.06
Tb.Nd (mm^−3^)	0.12 ± 0.01	0.24 ± 0.04	0.0003 ***
Tb.Tm (mm^−3^)	0.13 ± 0.03	0.16 ± 0.04	0.42
NdNdB (mm^−3^)	0.11 ± 0.02	0.18 ± 0.04	0.009 **
NdTmB (mm^−3^)	0.14 ± 0.03	0.16 ± 0.04	0.40
**Cortical Bone** **Morphometric Parameters**	**SLB (n = 6)** **Mean ± SD**	**MA (n = 6)** **Mean ± SD**	**Two-sample *t*-test** ***p*-value**
Ct.A.F (%)	86.4 ± 1.3	89.7 ± 2.76	0.08
Ct.Th (mm)	1.61± 0.15	0.98 ± 0.17	0.0001 ***
Ct.Po (%)	13.6 ± 1.1	10.23 ± 2.75	0.08

Level of significance: α = 0.05 (* *p*-value < 0.05; ** *p*-value < 0.001; *** *p*-value < 0.0001). SD—Standard Deviation.

**Table 8:** Results of the comparative microstructural analysis of the trabecular and cortical bone morphometric parameters of the experimental scapular acromion and mandibular mental protuberance bone samples.

**Trabecular Bone** **Morphometric Parameters**	**SAP (n = 6)** **Mean ± SD**	**MP (n = 6)** **Mean ± SD**	**Two-Sample *t*-Test** ***p*-Value**
Tb.BSA (mm^−1^)	12.13 ± 2.12	12.22 ± 2.04	0.94
BVF (%)	22.01± 4.88	23.6 ± 5.32	0.6
Tb.Po (%)	79.41 ± 2.51	77.85 ± 4.02	0.44
Tb.Th (mm)	0.16 ± 0.02	0.16 ± 0.02	0.91
Tb.N (mm^−1^)	0.9 ± 0.04	0.86 ± 0.06	0.30
Tb.Sp (mm)	0.84 ± 0.06	0.81 ± 0.05	0.51
Tb.Nd (mm^−3^)	0.12 ± 0.01	0.23 ± 0.03	0.92
Tb.Tm (mm^−3^)	0.13 ± 0.03	0.12 ± 0.02	0.83
NdNdB (mm^−3^)	0.11 ± 0.02	0.12 ± 0.02	0.29
NdTmB (mm^−3^)	0.14 ± 0.03	0.13 ± 0.01	0.66
**Cortical Bone** **Morphometric Parameters**	**SLB (n = 6)** **Mean ± SD**	**MA (n = 6)** **Mean ± SD**	**Two-Sample *t*-test** ***p*-value**
Ct.A.F (%)	86.4 ± 1.3	89.5 ± 2.05	0.52
Ct.Th (mm)	1.61± 0.15	1.96 ± 0.15	0.002 **
Ct.Po (%)	13.6 ± 1.1	9.6 ± 2.5	0.42

Level of significance: α = 0.05 (* *p*-value < 0.05; ** *p*-value < 0.001; *** *p*-value < 0.0001). SD—Standard Deviation.

## Data Availability

The original contributions presented in the study are included in the article/Supplementary Material, further inquiries can be directed to the corresponding author.

## Supplementary Materials

The following supporting information can be downloaded at https://www.mdpi.com/article/10.3390/jfb15120386/s1, Figure S1: Comparative analysis of the trabecular and cortical bone morphometric parameters of the experimental scapular coracoid and mandibular condyle bone samples. Figure S2: Comparative analysis of the trabecular and cortical bone morphometric parameters of the experimental scapular lateral border and mandibular angle bone samples. Figure S3: Comparative analysis of the trabecular and cortical bone morphometric parameters of the experimental scapular lateral border and mandibular mental protuberance bone samples. Figure S4: Comparative analysis of the trabecular and cortical bone morphometric parameters of the experimental scapular acromion and mandibular angle bone samples. Figure S5: Comparative analysis of the trabecular and cortical bone morphometric parameters of the experimental scapular acromion and mandibular mental protuberance bone samples.
