# Supplementary material for: Microstructural Analysis of the Human Scapula: Mandibular Bone Tissue Engineering Perspectives
Source: J Funct Biomater. 2024 Dec 20;15(12):386. doi: 10.3390/jfb15120386 (PMC11678577; doi:10.3390/jfb15120386)
Supplement: Supplementary file 1 [file jfb-15-00386-s001.zip › jfb-3349590-supplementary.pdf]

Supplementary Materials

Figure S1 Comparative analysis of the trabecular and cortical bone morphometric parameters of the experimental **scapular coracoid** and **mandibular condyle** bone samples

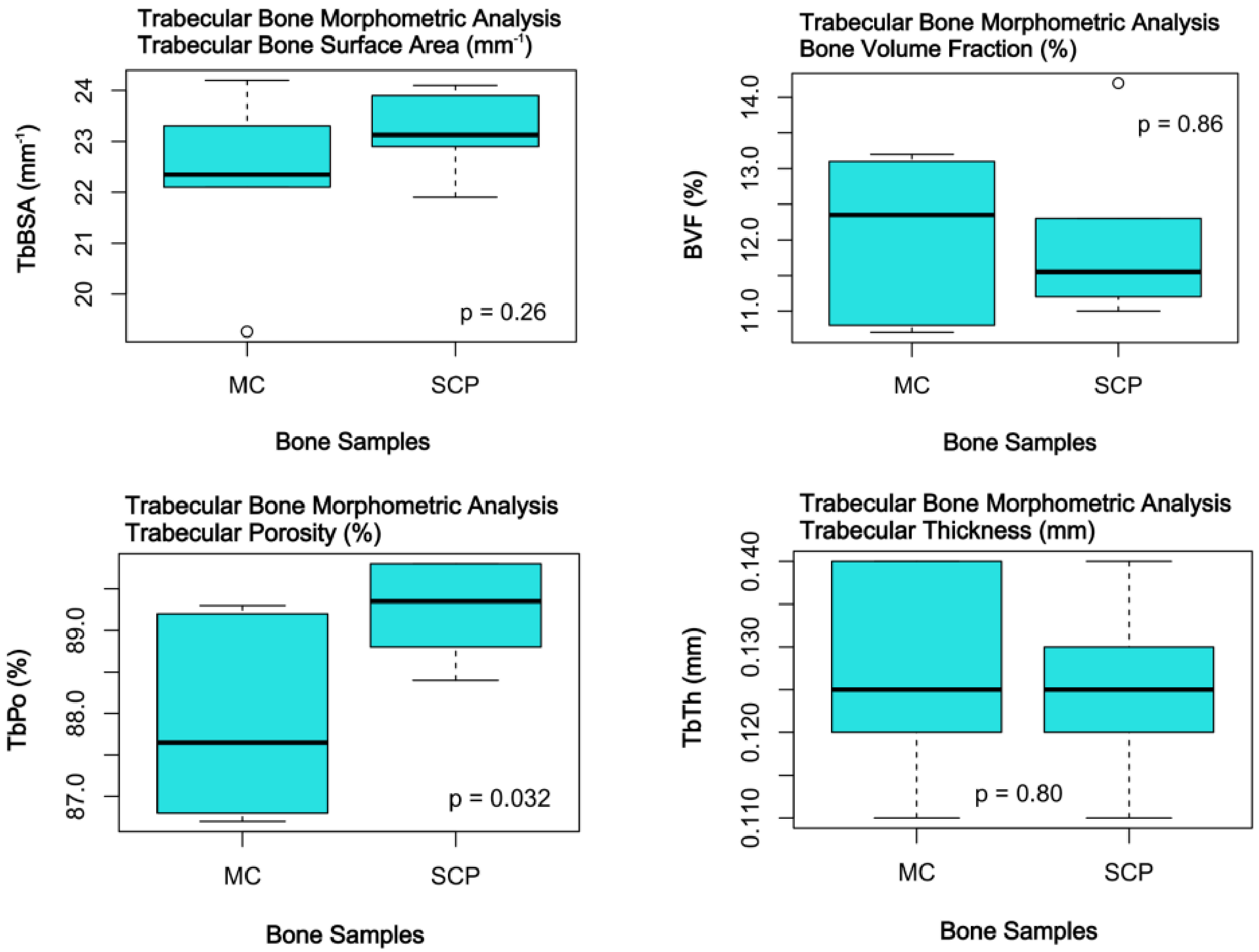

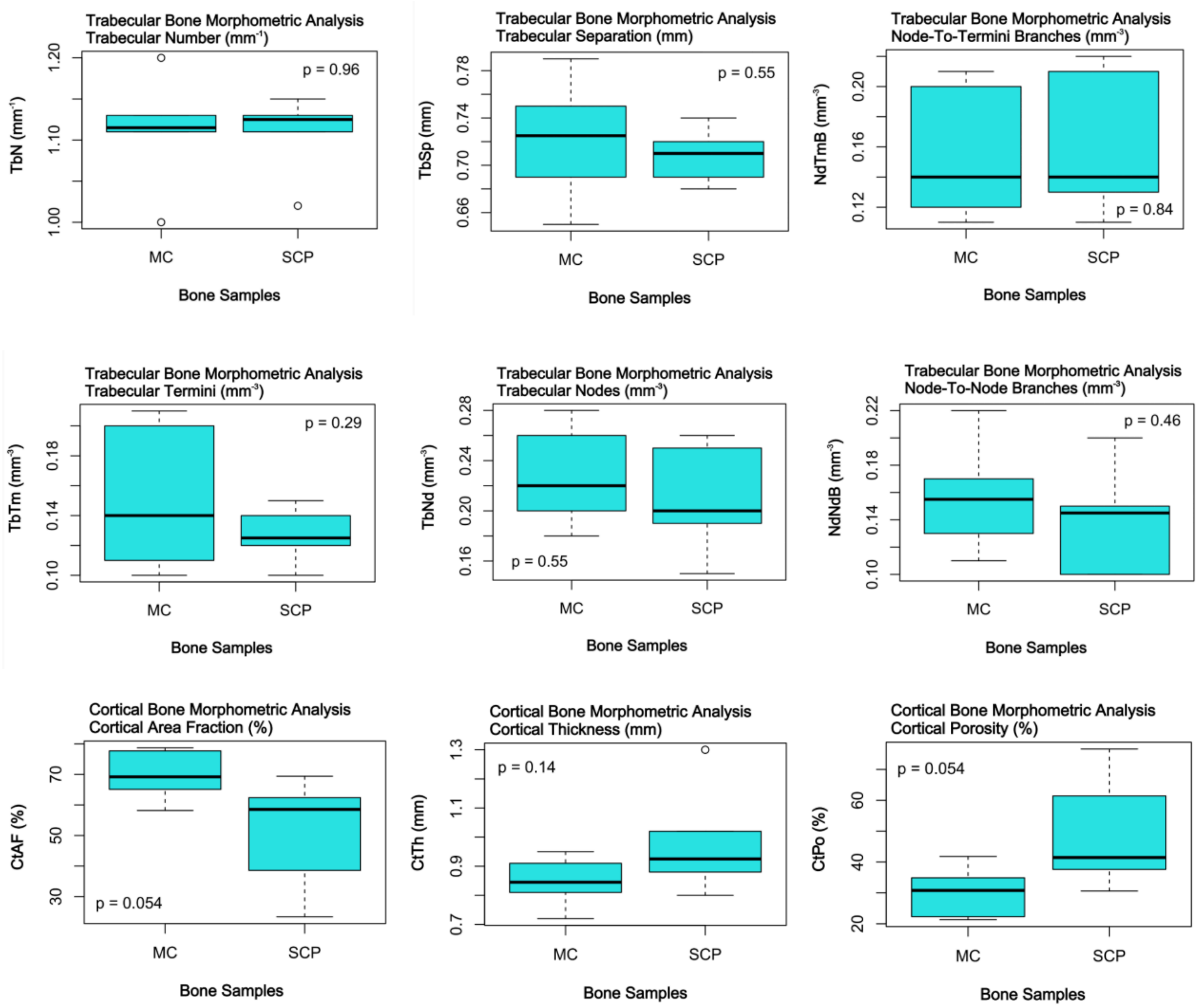
Trabecular Bone Morphometric Analysis
Trabecular Number (mm^{-1})
TbN (mm^{-1})
p = 0.96
Trabecular Bone Morphometric Analysis
Trabecular Separation (mm)
TbSp (mm)
p = 0.55
Trabecular Bone Morphometric Analysis
Node-To-Termini Branches (mm^{-3})
NdTmB (mm^{-3})
p = 0.84
Trabecular Bone Morphometric Analysis
Trabecular Termini (mm^{-3})
TbTm (mm^{-3})
p = 0.29
Trabecular Bone Morphometric Analysis
Trabecular Nodes (mm^{-3})
TbNd (mm^{-3})
p = 0.55
Trabecular Bone Morphometric Analysis
Node-To-Node Branches (mm^{-3})
NdNdB (mm^{-3})
p = 0.46
Cortical Bone Morphometric Analysis
Cortical Area Fraction (%)
CtAF (%)
p = 0.054
Cortical Bone Morphometric Analysis
Cortical Thickness (mm)
CtTh (mm)
p = 0.14
Cortical Bone Morphometric Analysis
Cortical Porosity (%)
CtPo (%)
p = 0.054
MC
SCP
Bone Samples

Figure S2 Comparative analysis of the trabecular and cortical bone morphometric parameters of the experimental **scapular lateral border** and **mandibular angle** bone samples

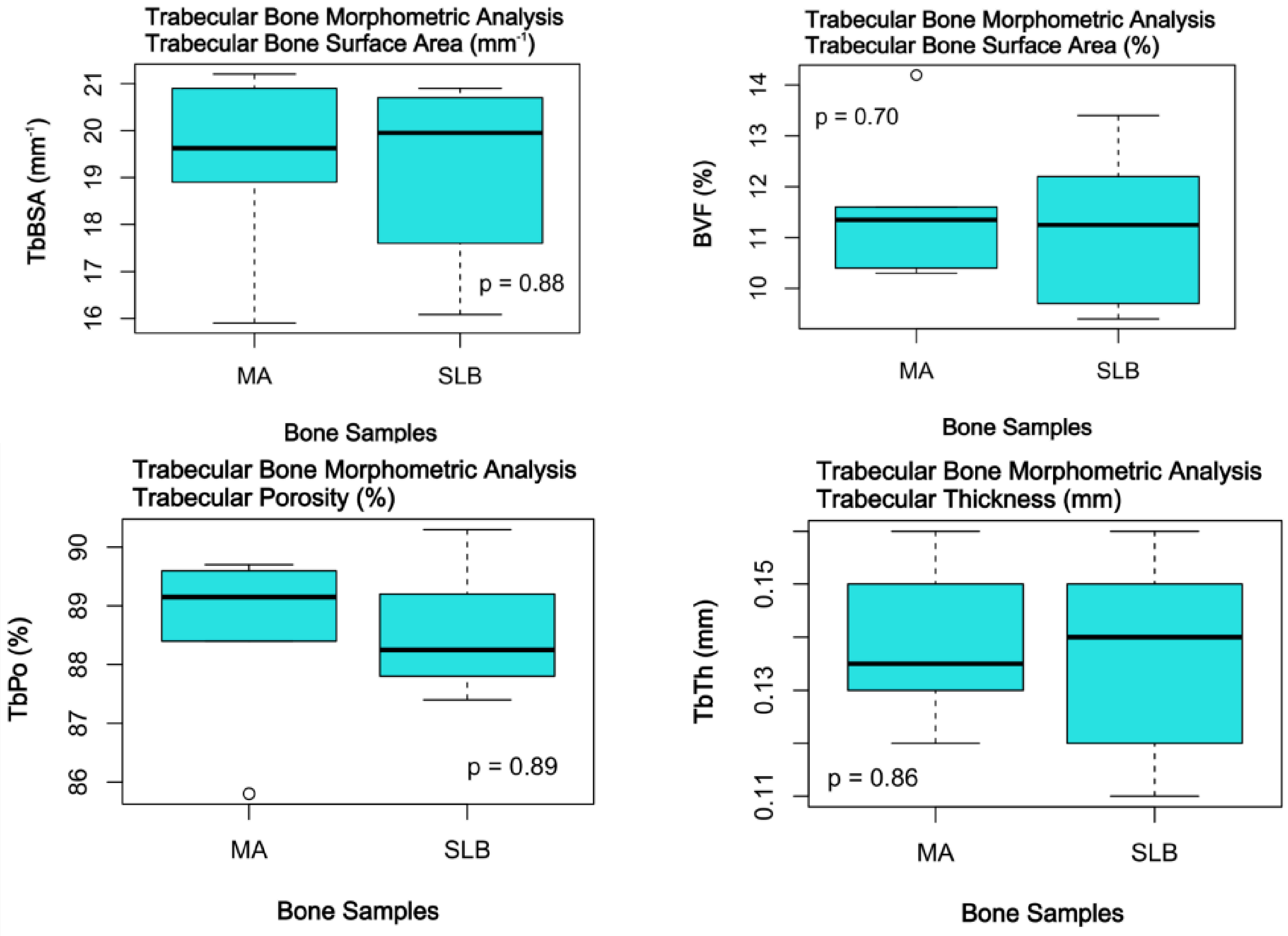

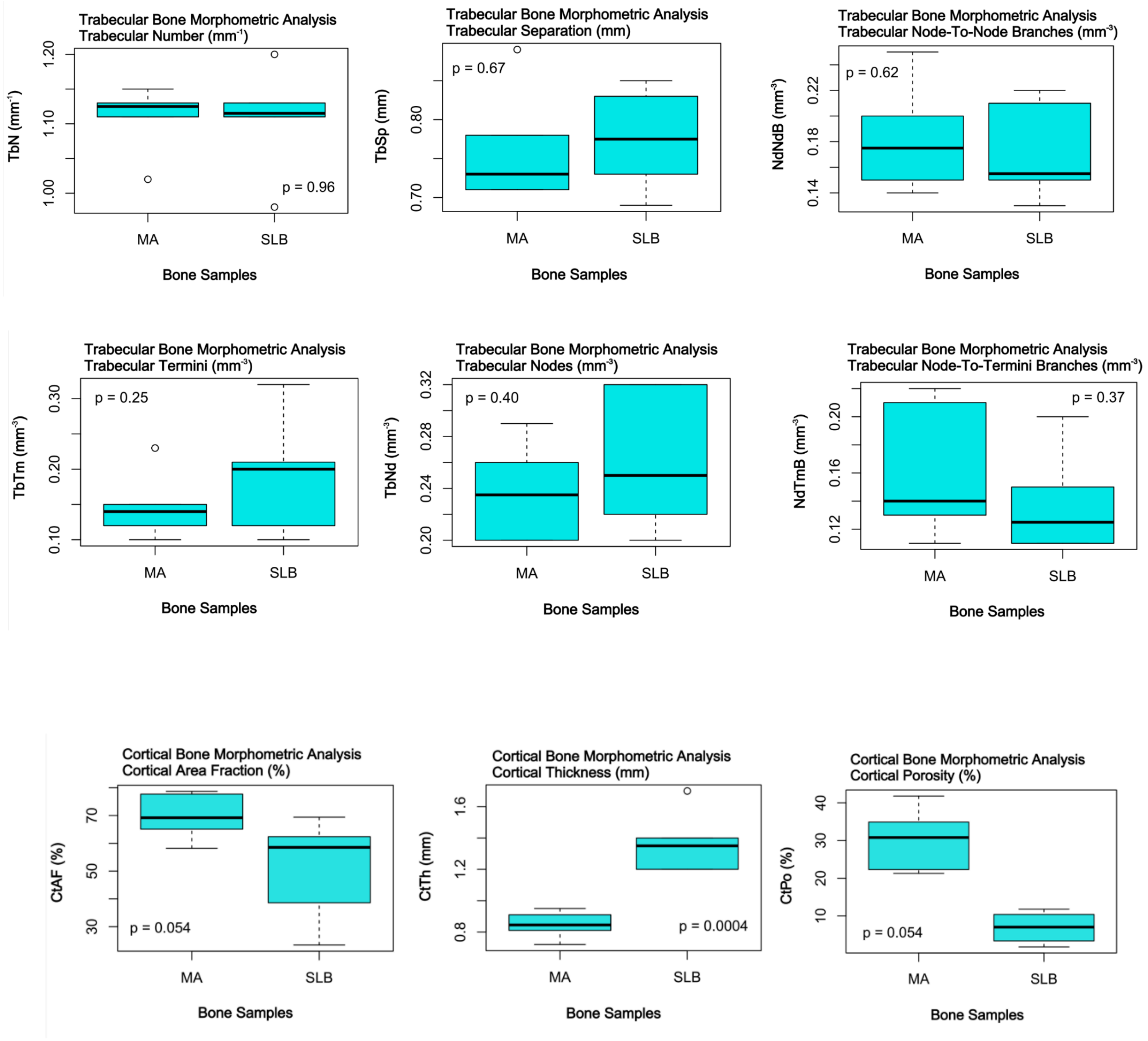

Trabecular Bone Morphometric Analysis
Trabecular Number (mm⁻¹)
TbN (mm⁻¹)
1.20
1.10
1.00
p = 0.96
MA
SLB
Bone Samples
Trabecular Bone Morphometric Analysis
Trabecular Separation (mm)
TbSp (mm)
0.80
0.70
p = 0.67
MA
SLB
Bone Samples
Trabecular Bone Morphometric Analysis
Trabecular Node-To-Node Branches (mm⁻³)
NdNdB (mm⁻³)
0.22
0.18
0.14
p = 0.62
MA
SLB
Bone Samples
Trabecular Bone Morphometric Analysis
Trabecular Termini (mm⁻³)
TbTm (mm⁻³)
0.30
0.20
0.10
p = 0.25
MA
SLB
Bone Samples
Trabecular Bone Morphometric Analysis
Trabecular Nodes (mm⁻³)
TbNd (mm⁻³)
0.32
0.28
0.24
0.20
p = 0.40
MA
SLB
Bone Samples
Trabecular Bone Morphometric Analysis
Trabecular Node-To-Termini Branches (mm⁻³)
NdTmB (mm⁻³)
0.20
0.16
0.12
p = 0.37
MA
SLB
Bone Samples
Cortical Bone Morphometric Analysis
Cortical Area Fraction (%)
CtAF (%)
70
50
30
p = 0.054
MA
SLB
Bone Samples
Cortical Bone Morphometric Analysis
Cortical Thickness (mm)
CtTh (mm)
1.6
1.2
0.8
p = 0.0004
MA
SLB
Bone Samples
Cortical Bone Morphometric Analysis
Cortical Porosity (%)
CtPo (%)
40
30
20
10
p = 0.054
MA
SLB
Bone Samples

Figure S3 Comparative analysis of the trabecular and cortical bone morphometric parameters of the experimental **scapular lateral border** and **mandibular mental protuberance** bone samples

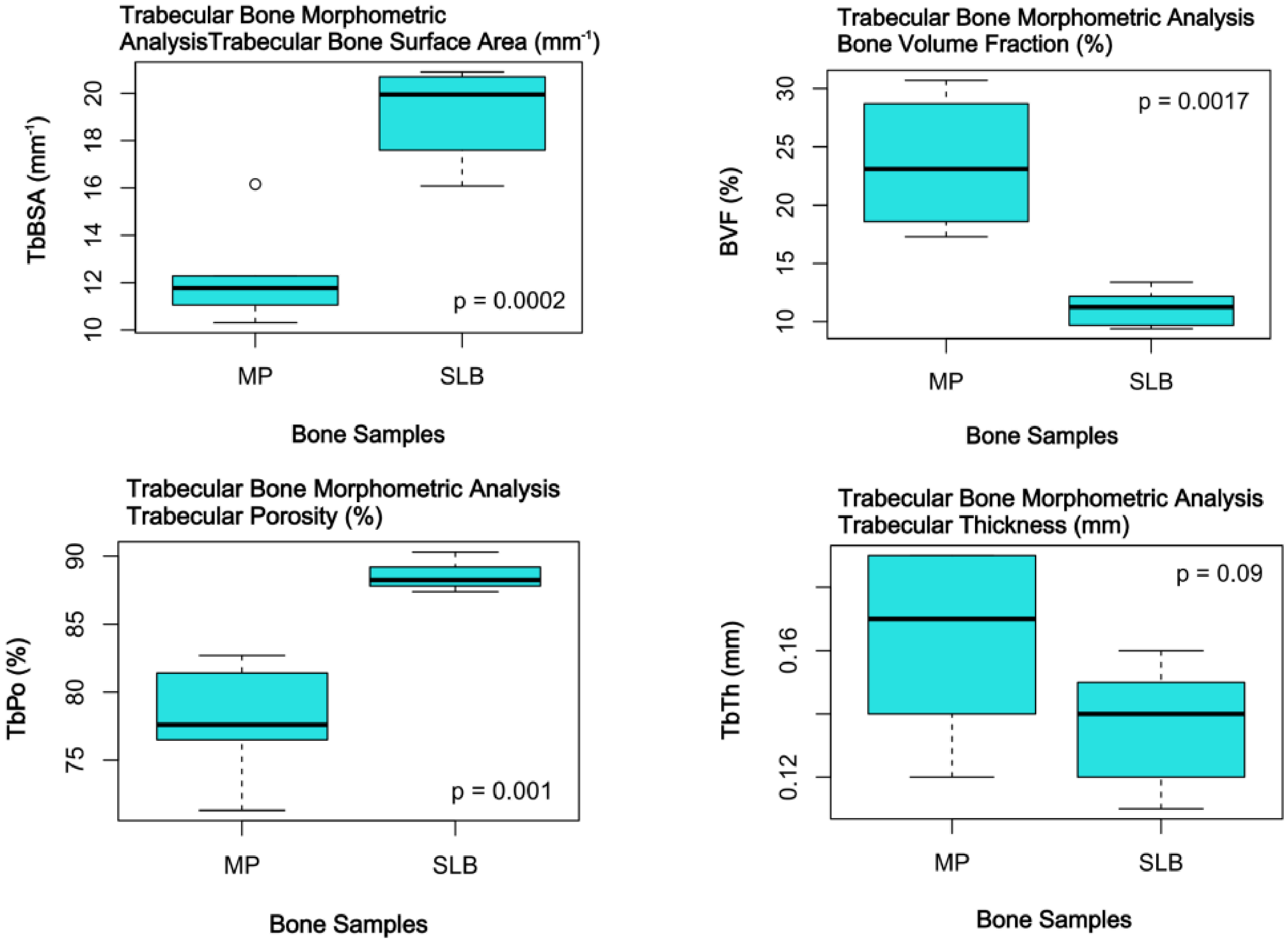

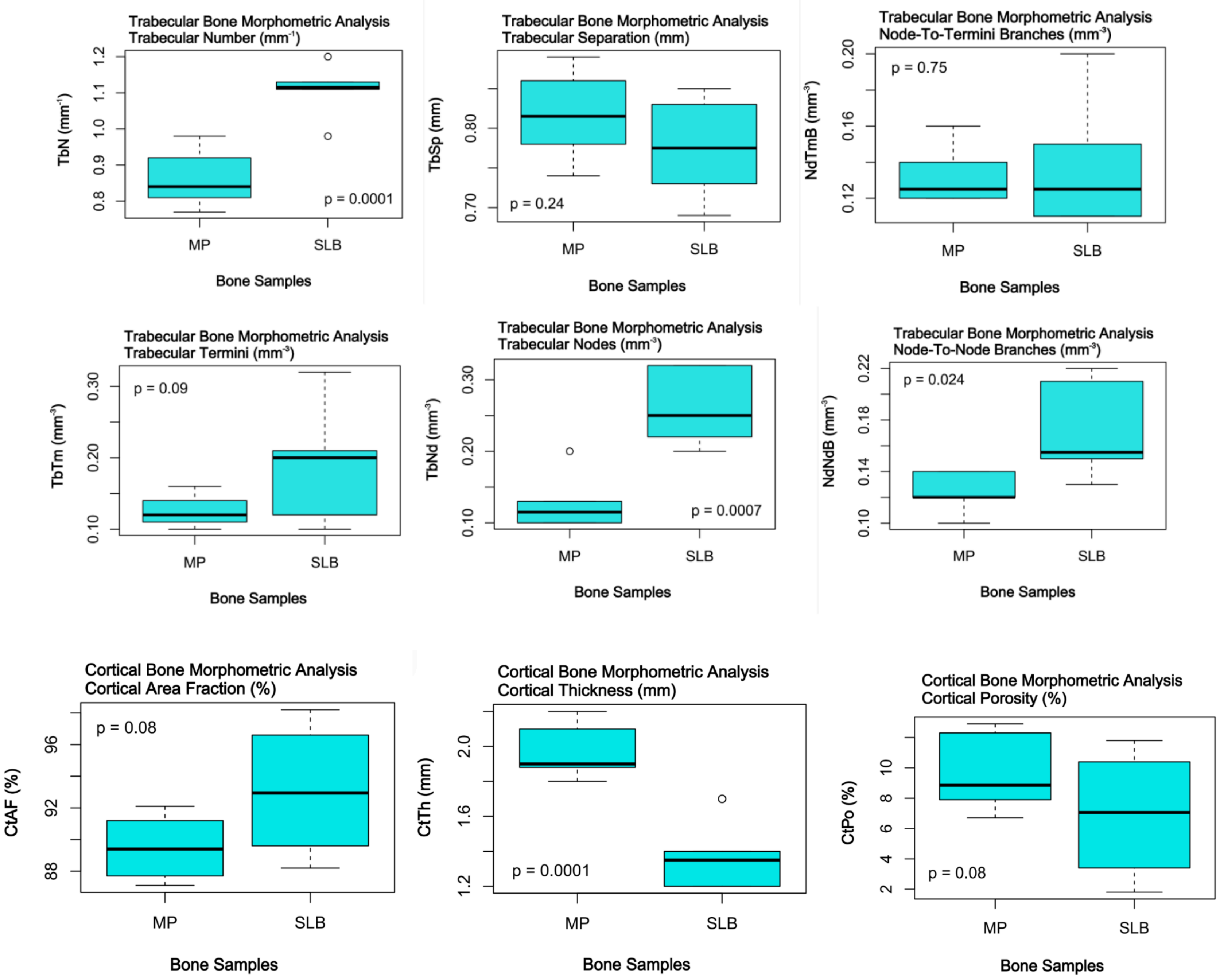
Trabecular Bone Morphometric Analysis
Trabecular Number (mm^{-1})
TbN (mm^{-1})
0.8 0.9 1.0 1.1 1.2
p = 0.0001
MP SLB
Bone Samples
Trabecular Bone Morphometric Analysis
Trabecular Separation (mm)
TbSp (mm)
0.70 0.80
p = 0.24
MP SLB
Bone Samples
Trabecular Bone Morphometric Analysis
Node-To-Termini Branches (mm^{-3})
NdTmB (mm^{-3})
0.12 0.16 0.20
p = 0.75
MP SLB
Bone Samples
Trabecular Bone Morphometric Analysis
Trabecular Termini (mm^{-3})
TbTm (mm^{-3})
0.10 0.20 0.30
p = 0.09
MP SLB
Bone Samples
Trabecular Bone Morphometric Analysis
Trabecular Nodes (mm^{-3})
TbNd (mm^{-3})
0.10 0.20 0.30
p = 0.0007
MP SLB
Bone Samples
Trabecular Bone Morphometric Analysis
Node-To-Node Branches (mm^{-3})
NdNdB (mm^{-3})
0.10 0.14 0.18 0.22
p = 0.024
MP SLB
Bone Samples
Cortical Bone Morphometric Analysis
Cortical Area Fraction (%)
CtAF (%)
88 92 96
p = 0.08
MP SLB
Bone Samples
Cortical Bone Morphometric Analysis
Cortical Thickness (mm)
CtTh (mm)
1.2 1.6 2.0
p = 0.0001
MP SLB
Bone Samples
Cortical Bone Morphometric Analysis
Cortical Porosity (%)
CtPo (%)
2 4 6 8 10
p = 0.08
MP SLB
Bone Samples

Figure S-4 Comparative analysis of the trabecular and cortical bone morphometric parameters of the experimental **scapular acromion** and **mandibular angle** bone samples

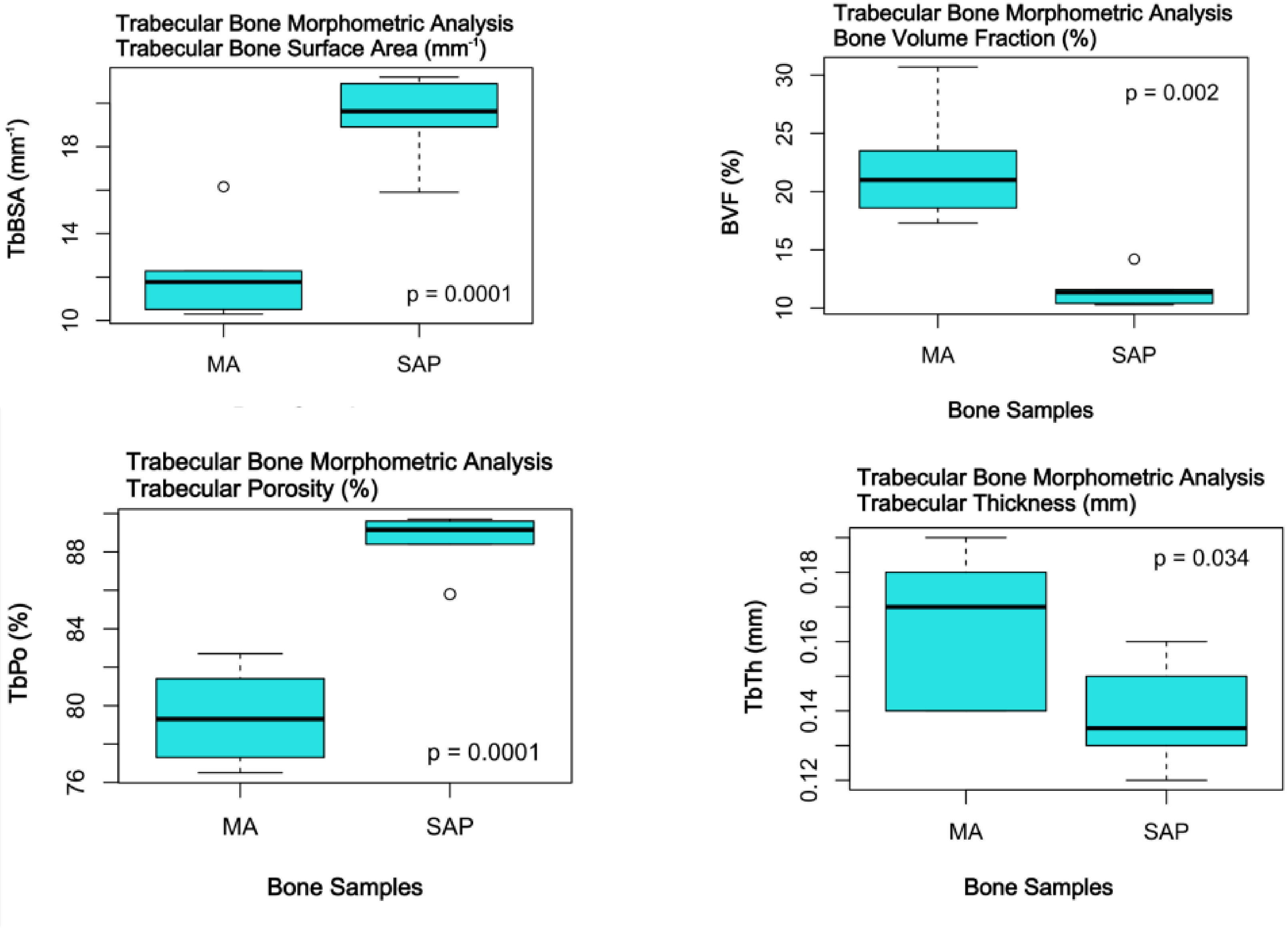

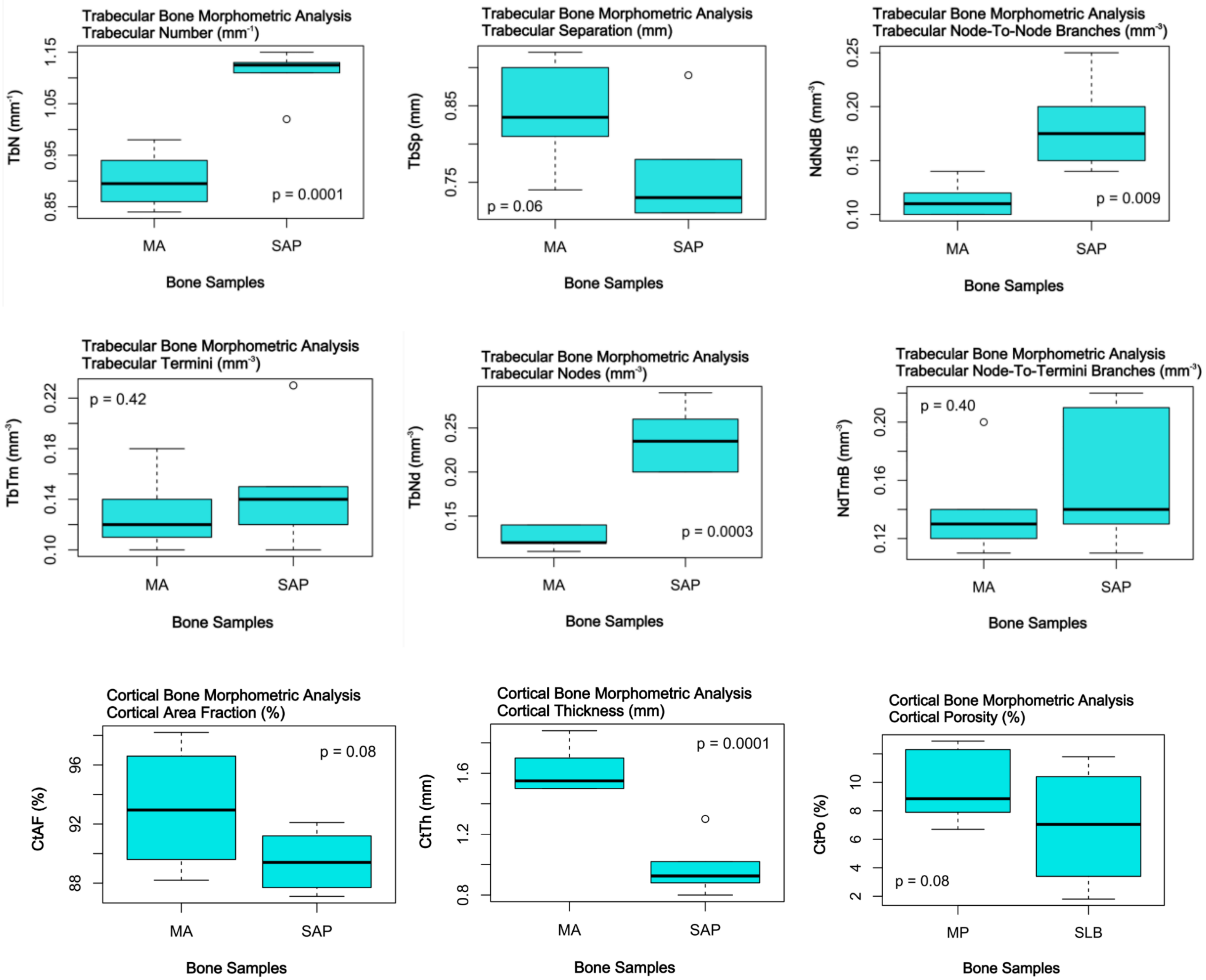
Trabecular Bone Morphometric Analysis
Trabecular Number (mm-1)
TbN (mm-1)
p = 0.0001
Trabecular Bone Morphometric Analysis
Trabecular Separation (mm)
TbSp (mm)
p = 0.06
Trabecular Bone Morphometric Analysis
Trabecular Node-To-Node Branches (mm-3)
NdNdB (mm-3)
p = 0.009
Trabecular Bone Morphometric Analysis
Trabecular Termini (mm-3)
TbTm (mm-3)
p = 0.42
Trabecular Bone Morphometric Analysis
Trabecular Nodes (mm-3)
TbNd (mm-3)
p = 0.0003
Trabecular Bone Morphometric Analysis
Trabecular Node-To-Termini Branches (mm-3)
NdTmB (mm-3)
p = 0.40
Cortical Bone Morphometric Analysis
Cortical Area Fraction (%)
CtAF (%)
p = 0.08
Cortical Bone Morphometric Analysis
Cortical Thickness (mm)
CtTh (mm)
p = 0.0001
Cortical Bone Morphometric Analysis
Cortical Porosity (%)
CtPo (%)
p = 0.08
MA
SAP
MP
SLB
Bone Samples

Figure S5 Comparative analysis of the trabecular and cortical bone morphometric parameters of the experimental **scapularacromion** and **mandibular mental protuberance** bone samples

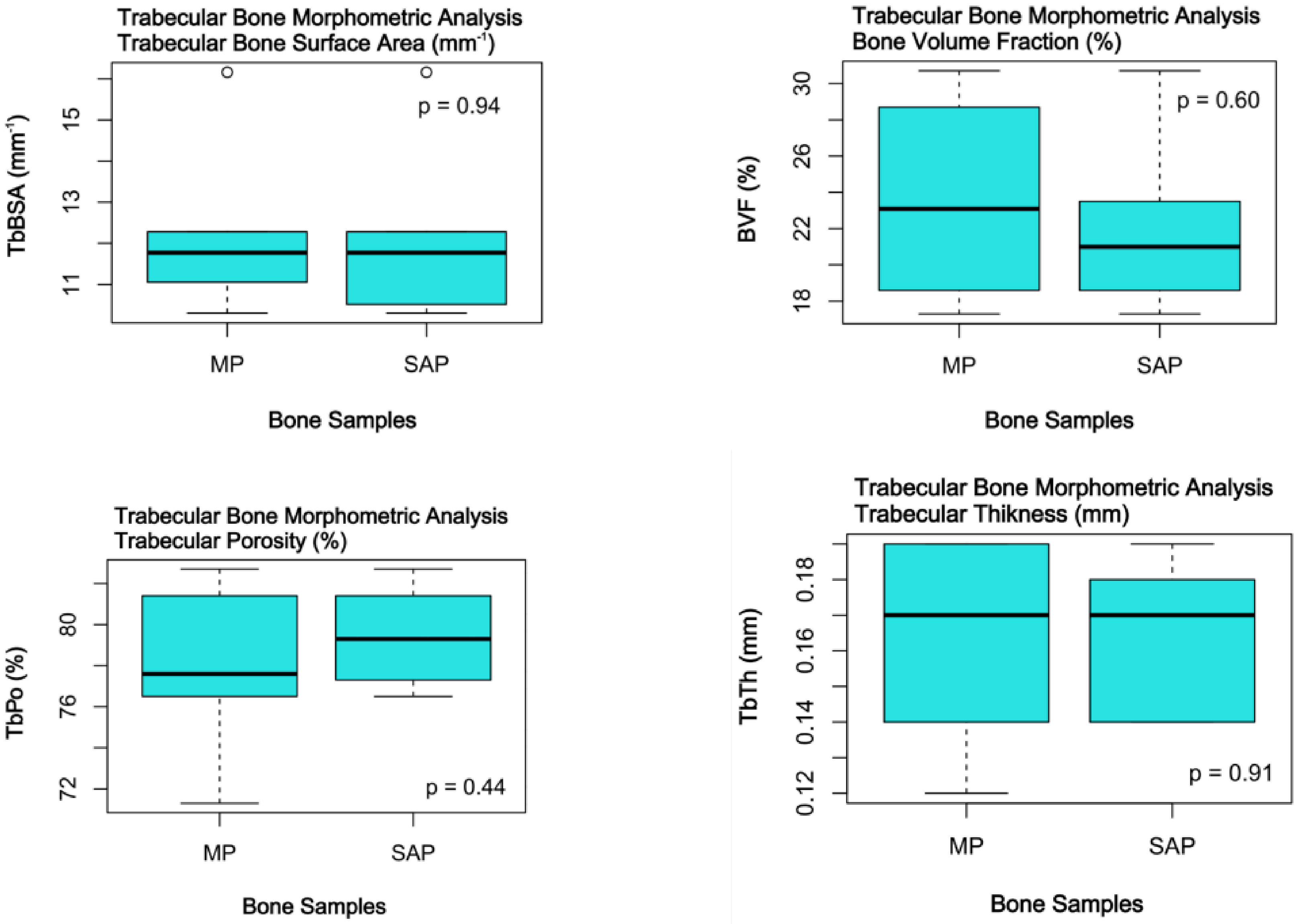

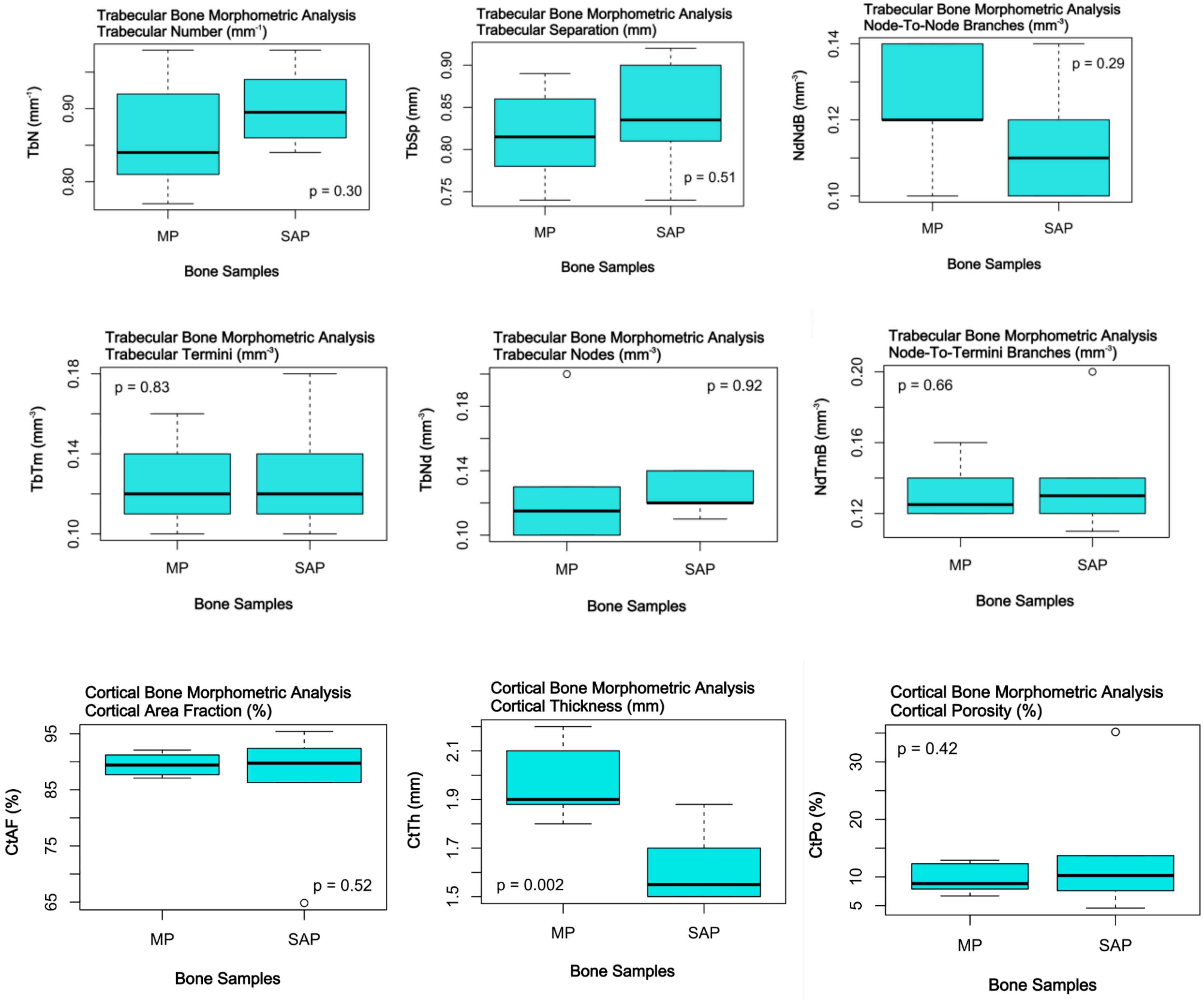
Trabecular Bone Morphometric Analysis
Trabecular Number (mm^{-1})
TbN (mm^{-1})
0.80
0.90
p = 0.30
MP
SAP
Bone Samples
Trabecular Bone Morphometric Analysis
Trabecular Separation (mm)
TbSp (mm)
0.75
0.80
0.85
0.90
p = 0.51
MP
SAP
Bone Samples
Trabecular Bone Morphometric Analysis
Node-To-Node Branches (mm^{-3})
NdNdB (mm^{-3})
0.10
0.12
0.14
p = 0.29
MP
SAP
Bone Samples
Trabecular Bone Morphometric Analysis
Trabecular Termini (mm^{-3})
TbTm (mm^{-3})
0.10
0.14
0.18
p = 0.83
MP
SAP
Bone Samples
Trabecular Bone Morphometric Analysis
Trabecular Nodes (mm^{-3})
TbNd (mm^{-3})
0.10
0.14
0.18
p = 0.92
MP
SAP
Bone Samples
Trabecular Bone Morphometric Analysis
Node-To-Termini Branches (mm^{-3})
NdTmB (mm^{-3})
0.12
0.16
0.20
p = 0.66
MP
SAP
Bone Samples
Cortical Bone Morphometric Analysis
Cortical Area Fraction (%)
CtAF (%)
65
75
85
95
p = 0.52
MP
SAP
Bone Samples
Cortical Bone Morphometric Analysis
Cortical Thickness (mm)
CtTh (mm)
1.5
1.7
1.9
2.1
p = 0.002
MP
SAP
Bone Samples
Cortical Bone Morphometric Analysis
Cortical Porosity (%)
CtPo (%)
5
10
20
30
p = 0.42
MP
SAP
Bone Samples
